# LSD1 Facilitates Pro-Inflammatory Polarization of Macrophages by Repressing Catalase

**DOI:** 10.3390/cells10092465

**Published:** 2021-09-18

**Authors:** Maciej Sobczak, Magdalena Strachowska, Karolina Gronkowska, Iwona Karwaciak, Łukasz Pułaski, Agnieszka Robaszkiewicz

**Affiliations:** 1 Department of General Biophysics, Faculty of Biology and Environmental Protection, University of Lodz, Pomorska 141/143, 90-236 Lodz, Poland; maciej.sobczak@edu.uni.lodz.pl (M.S.); magdalena.strachowska@edu.uni.lodz.pl (M.S.); karolina.gronkowska@edu.uni.lodz.pl (K.G.); 2 Laboratory of Transcriptional Regulation, Institute of Medical Biology PAS, Lodowa 106, 93-232 Lodz, Poland; isachrajda@cbm.pan.pl (I.K.); lpulaski@biol.uni.lodz.pl (Ł.P.); 3 Department of Molecular Biophysics, Faculty of Biology and Environmental Protection, University of Lodz, Pomorska 141/143, 90-236 Lodz, Poland

**Keywords:** macrophages, pro-inflammatory markers, catalase, LSD1, gene transcription

## Abstract

The increased level of hydrogen peroxide accompanies some modes of macrophage specification and is linked to ROS-based antimicrobial activity of these phagocytes. In this study, we show that activation of toll-like receptors with bacterial components such as LPS is accompanied by the decline in transcription of hydrogen peroxide decomposing enzyme-catalase, suppression of which facilitates the polarization of human macrophages towards the pro-inflammatory phenotype. The chromatin remodeling at the *CAT* promoter involves LSD1 and HDAC1, but activity of the first enzyme defines abundance of the two proteins on chromatin, histone acetylation status and the *CAT* transcription. LSD1 inhibition prior to macrophage activation with LPS prevents *CAT* repression by enhancing the LSD1 and interfering with the HDAC1 recruitment to the gene promoter. The maintenance of catalase level with LSD1 inhibitors during M1 polarization considerably limits LPS-triggered expression of some pro-inflammatory cytokines and markers such as *IL1β*, *TNFα*, *COX2*, *CD14*, *TLR2*, and *IFNAR*, but the effect of LSD1 inhibitors is lost upon catalase deficiency. Summarizing, activity of LSD1 allows for the *CAT* repression in LPS stimulated macrophages, which negatively controls expression of some key pro-inflammatory markers. LSD1 inhibitors can be considered as possible immunosuppressive drugs capable of limiting macrophage M1 specialization.

## 1. Introduction

Elevated levels of hydrogen peroxide have been reported in various cell types inter alia in some cancer cells and pro-inflammatory—M1 macrophages. In phagocytes, it acts as a pathogen-killing agent when converted to hypochlorous acid by myeloperoxidase, as an intra- and cell-to-cell signaling molecule promoting TNFα and nitric oxide release by adjacent cells [1]. Excessive production of hydrogen peroxide (H_2_O_2_) in M1 macrophages is majorly assigned to NADPH oxidase(s), which become active in response to stimulation of toll-like receptors (TLRs). The hyperreactive superoxide anion released by NADPH oxidese(s) is immediately converted to more stable and membrane permeable molecule—H_2_O_2_. Some types of macrophage polarization, tumorigenesis, and also transformation of healthy cells with T-antigen of SV40 or Ras lead to considerable decline in the catalase level, which may help to maintain increased H_2_O_2_ concentration inside cells. Our previous reports showed that stimulation of TLR4 in mouse bone marrow-derived macrophages with LPS caused Cat repression, but increased transcription of Sod2, Gsr, Txnrd1, and Prdx1 [2]. A similar effect was also observed in a monocyte-to-macrophage differentiation model of THP1 and Mono Mac 6, monocytic cell lines, where the cells were first differentiated with phorbol 12-myristate 13-acetate and then polarized with TLR1/2 ligand Pam3CSK4, a synthetic triacylated lipopeptide (LP) that mimics the acylated amino terminus of bacterial lipopeptides [3]. Both TLR ligands act as potent activators of the pro-inflammatory transcription factor NF-κB. As long as LPS triggers M1, the pro-inflammatory phenotype, Pam3CSK4 is documented to stimulate the cells into a solid M1 or M2b—immunoregulative subtypes of anti-inflammatory macrophages [4,5]. The meaning of catalase decline in the (patho)physiology of macrophages was confirmed by genetic engineering that allowed for overexpression of the enzyme. Transgenic mice with myeloid lineage cell-specific overexpression of catalase (TgCat-MLC mice) showed deficient neovascularization, decreased macrophage infiltration of ischemic tissues and macrophage migration in vitro, and lower mRNA levels of inflammatory markers, such as tumor necrosis factor-α, osteopontin, and matrix mettaloproteinase-9, thereby indicating the role of the oxidant in regulating macrophages’ ability to infiltrate ischemic tissues [6]. Redox-relevant enzymes also control macrophage pro-inflammatory phenotype and M1 response to damaging agents, as was reported in our recent paper, where inhibition of termination of *SOD2* transcription upon TLR4 activation increased the protein level of the enzyme and rendered pro-inflammatory macrophages even more resistant to oxidative stress [7].

The intentional interference or enhancement of macrophage development and differentiation, polarization, or response to damaging stimuli via up- or downregulation of particular antioxidant enzymes might be of great importance for the future therapies of diseases, where the initiation or propagation step involves innate immune responses. These include but are not limited toatherosclerosis, rheumatoid arthritis, amyotrophic lateral sclerosis, or multiple sclerosis [8,9,10]. To target expression of antioxidant enzymes for immunomodulation the control of their turnover must be disclosed. The underlying mechanisms of *CAT* downregulation during pro-inflammatory macrophage specialization are still unknown. In cancer cells, *CAT* repression has been shown to be linked to hypermethylation of the CpG island in the gene promoter, to histone deacetylation, to the expression of miR-30b, and to the high level of c-abl, which can lead to catalase protein degradation via post-translational modifications [11]. The lack of any information on the possible mechanism of *CAT* repression during macrophage M1 polarization prompted us to study the epigenetic changes at the promoter of this gene upon stimulation of granulocyte–macrophage colony-stimulating factor (GM-CSF)-differentiated macrophages (M0) with pro-inflammatory TLR4 ligand—bacterial endotoxin (LPS).

In this paper, we described contribution of lysine-specific histone demethylase 1A (KDM1A or LSD1) to LPS-induced catalase suppression. This allowed us to test the LSD1 inhibitors as compounds capable of the catalase maintenance in pro-inflammatory macrophages and to study the possible catalase involvement in the M1 polarization. The two studied LSD1 inhibitors—SP2509 and GSK-LSD1—emerged as possible immunosuppressive and anti-inflammatory drugs capable of declining transcription of pro-inflammatory cytokines and M1-relevant surface markers by preventing CAT repression in LPS-stimulated macrophages. Moreover, we disclose a new regulatory mode of LSD1 and HDAC1 functional interaction, where LSD1 activity orchestrates the recruitment of both enzymes to the *CAT* promoter.

## 2. Materials and Methods

### 2.1. Chemicals and Reagents

RosetteSep™ Monocyte Enrichment Cocktail was purchased from STEMCELL Technologies (Grenoble, France), cell culture media were from Biowest (CytoGen, Zgierz, Poland), granulocyte–macrophage colony-stimulating factor (GM-CSF) from PeproTech (London, UK), anti-rabbit IgG (whole molecule) (A0545) and anti-mouse IgG (whole molecule) (A4416) peroxidase-labeled antibodies produced in goat, BLUeye prestained protein marker (#94964), SIGMAFAST™ Protease Inhibitor Tablets (PIC), sodium butyrate (iHDAC), C646 (iEP300), ML385 (iNRF2) and oligonucleotides for real-time PCR were from Sigma-Aldrich (Poznan, Poland). Dynabeads™ Protein G, High-Capacity cDNA Reverse Transcription Kit, Click-iT™ Nascent RNA Capture Kit, SuperSignal™ West Pico Chemiluminescent Substrate, TRI Reagent™, PowerUp™ SYBR^®^ Green Master Mix, and anty-HDAC1 (PA1-860) were from Thermofisher Scientific (Thermofisher Scientific, Warsaw, Poland). Advanced TC™ culture plates, SP2509 (iLSD1a), GSK-LSD1 (iLSD1b) and PBIT (iKDM5B) were from Greiner Bio-One and Cayman Chemical (Biokom, Janki/Warsaw, Poland). KAPA HiFi™ HotStart ReadyMix (2×) from KapaBiosystems was purchased from Polgen (Łódź, Poland), while EvaGreen^®^ Dye, 20× in water, from Biotium (Corporate Place, Hayward, CA, USA). Human Toll-like Receptor Signaling Primer Library (HTLR-I) and Human Cytokine/Chemokine Receptor Primer Library I (HCCR-I) were purchased from RealTimePrimers.com (Prospecta, Warsaw, Poland).

ChIP-grade antibodies: anti-LSD1 (#2139), anti-histone H3 (#4620), anti-H3K27ac (#4353), anti-H3K4me3 (#9751), anti-H3K4me1 (#5326), anti-NRF2 (#12721) and normal rabbit IgG (#2729) were purchased from Cell Signaling Technology (LabJOT, Warsaw, Poland). Anti-CAT ((H-9): sc-271803) antibody, siCAT (sc-45330), siLSD1 (sc-60970) were from Santa Cruz Biotechnology (AMX, Lodz, Poland).

### 2.2. Monocyte Isolation, Differentiation to Macrophages, and Stimulation with TLR Ligands

Buffy coats of healthy donors were purchased from Blood Bank in Lodz, Poland. Monocytes were isolated using RosetteSep Monocyte Enrichment Cocktail as described previously, allowed to attach to Advanced TC™ culture plates in RPMI medium supplemented with 10% FBS, penicillin/streptomycin (50 U/mL and 50 µg/mL, respectively), and then differentiated into macrophages (M0) with GM-CSF (10 ng/mL) for 6 days. Macrophages were treated with LPS (10 ng/mL) for 24 h.

The inhibitors of chromatin remodeling enzymes were added before stimulation at the following doses: C646 (iEP300), 10 µM; sodium butyrate (iHDAC), 200 µM; SP2509 (iLSD1a), 0.1 µM; GSK-LSD1 (iLSD1b), 0.1 µM; and PBIT (iKDM5B), 5 µM.

### 2.3. Gene Silencing

Catalase and LSD1 were transiently silenced, as described previously [12]. In brief, 100,000 cells were incubated with siRNA:Lipofectamine RNAiMAX complexes in OptiMEM for 28 h. A non-targeting siRNA was used as a control (siCTRL). The silencing was confirmed by real-time PCR and/or Western blot.

### 2.4. Evaluation of CAT and M1 Markers Expression

Total RNA was isolated with TRI Reagent™ and reverse transcribed with the High-Capacity cDNA Reverse Transcription Kit as described previously. mRNA of CAT and other genes was quantified in real-time PCR reaction with the following primers (Table 1).

Primers for CD14, TLR2, STAT1, CXCL9, IFNAR2, IL1RN were taken from Human Toll-like Receptor Signaling Primer Library (HTLR-I) and Human Cytokine/Chemokine Receptor Primer Library I (HCCR-I).

PowerUp™ SYBR^®^ Green Master Mix was used according to the manufacturer instruction.

Ongoing transcription/de novo synthesis of mRNA was monitored with the Click-iT™ Nascent RNA Capture Kit as described by Wiśnik et al. [13]. Ethylene uridine (0.25 mM) was added to macrophages after LPS treatment for 6 h prior to cell lysis with TRI Reagent™.

In both approaches, CAT expression was normalized to the median of mRNA of ACTB, GAPDH, and B2M.

For catalase protein detection by Western blot, macrophage lysates were separated by SDS-PAGE, transferred to nitrocellulose membrane, blocked with 5% bovine serum albumin, and stained overnight with primary antibody in 1% bovine albumin in PBS-0.1% Tween at 4 °C. After staining with HRP-conjugated secondary antibody, the signal was developed using SuperSignal™ West Pico Chemiluminescent Substrate. Pictures were acquired by ChemiDoc-IT2 (UVP, Meranco, Poznan, Poland), and H3 was used as loading control.

### 2.5. Chromatin Immunoprecipitation

Chromatin immunoprecipitation was carried out according to a previously described protocol [13]. In brief, cells were fixed with 1% formaldehyde, and then lysed and sonicated with Bandelin Sonopuls (HD 2070) to obtain chromatin fragments of ~250–300 bp. Cell lysates were incubated with antibodies and Dynabeads protein G magnetic beads at 4 °C overnight. Immunoprecipitates were washed, and DNA was isolated with UltraPure™ Phenol/Chloroform/Isoamyl Alcohol (25:24:1, *v*/*v*); 10% cell lysate was taken as an input. For H3K27ac quantification, cells were added to with iHDAC (0.5 mM) prior to lysis, and the HDAC inhibitor was present at the indicated concentration in cell lysates during sample preparation. The CAT proximal promoter (region spanning the binding sites for transcription factors and chromatin remodeling enzymes according to UCSC Genome Browser) was amplified using KAPA HiFi™ HotStart ReadyMix supplemented with EvaGreen^®^ Dye and 0.5% DMSO, and the following primers: Fwd 5′-CTGGGTATCTCCGGTCTTCA-3′ and Rev 5′- GACTTCAGGCTCAGCCAATC-3′.

To confirm the enrichment of RNA polymerase II pausing complex in response to macrophage treatment with LPS we quantified the enrichment of HEXIM and NELF-E at the two CAT exons with the following primers: Fwd 5′-GCCTTTGGCTACTTTGAGGTC-3′ and Rev 5′-GTGGAGAACCGAACTGCGAT-3′ for exon 3, and Fwd 5′-TGCTCCAAATTACTACCCCAAC-3′ and Rev 5′-TGTTGAATCTCCGCACTTCTC3′ for exon 10.

### 2.6. Co-Immunoprecipitation

HDAC1 was immunoprecipitated with 5 µg of anti-HDAC1 antibody in the presence of Dynabeads protein G at 4 °C for 3 h, as described in detail by Sobczak et al. [12]. After washing on a magnetic stand, HDAC1-interacting proteins were separated by SDS-PAGE. LSD1 and RelA were detected by Western blot. Rabbit normal IgG served as an isotypic control in immunoprecipitation, and 10% cell lysate was taken as an input.

### 2.7. Statistical Analysis

Data are shown as mean ± standard deviation of the mean (SEM). One-way analysis of variance (ANOVA1) was carried out in GraphPad Prism 5 to compare means in several groups (marked with * when *p* < 0.05, ** when *p* < 0.01, *** when *p* < 0.001), and followed by Tukey post hoc test (marked with * when *p* < 0.05, ** when *p* < 0.01, *** when *p* < 0.001).

## 3. Results

### 3.1. TLR Ligands Reduce CAT Total mRNA Levels in Human Macrophages

**Figure 1:**
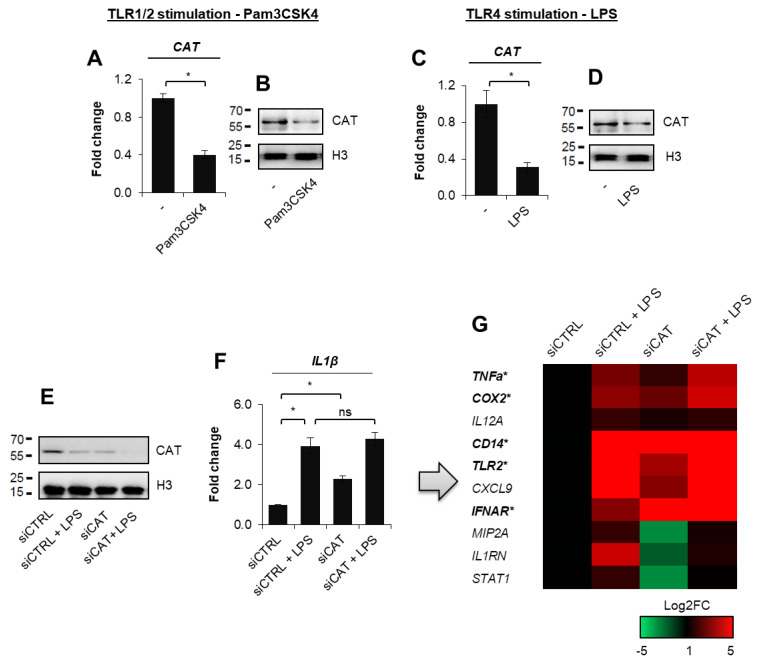
The decline in CAT total mRNA levels in human macrophages correlates with transcription of some macrophage pro-inflammatory markers. Granulocyte–macrophage colony-stimulating factor (GM-CSF)-differentiated human macrophages were quantified for CAT expression after their exposure to Pam3CSK4 (50 ng/mL; A and B) and LPS (10 ng/mL; C and D). mRNA level in (**A**,**C**) was measured by real-time PCR, while catalase protein in (**B**,**D**) was visualized by Western blot after 24 h of cell incubation with TLR ligands. To verify the possible effect of catalase deficiency on selected pro-inflammatory features, CAT was targeted with siRNA and the silencing was confirmed by Western blot vs. siRNA non-template control (siCTRL) 48 h after cell transfection (**E**). The mRNA level of M1 markers was quantified by real-time PCR and shown in bar chart for IL1β as an example (**F**) and heat map for other genes (**G**). Bolded genes marked with the star represent these substantially repressed by catalase in non-polarized cells. Western blots show representative images from three independent experiments. Bars in the figures represent mean ± standard error of the mean (SEM). One-way analysis of variance (ANOVA) was carried out in GraphPad Prism 5 to compare means in several groups and the differences between each two groups were tested with the Tukey post hoc test (marked with * when *p* < 0.05, with ns when *p* > 0.05).

Bearing in mind that pro-inflammatory polarization of mouse macrophages isolated from the bone marrow as well as monocytic THP1 and Mono Mac6 cancer cells cause substantial reduction in catalase expression, we tested first if the same effects could be observed in human macrophages derived from monocytes, which were incubated with GM-CSF for 6 days. Activation of TLR2 with Pam3CSK4 and TLR4 with LPS resulted in similar suppression of the mRNA level (Figure 1A,C) and in a decrease in the abundance of the enzyme (Figure 1B,D, Figure S1).

In search for physiological meaning of catalase decline during M1 polarization we checked if this enzyme is involved in the transcription control of pro-inflammatory cytokines and M1-specific factors. From the first group we chose for testing the typical pro-inflammatory mediators such as *IL1β*, *TNFα*, *COX2*, *MIP2A*, and *IL12A*, whereas from the second group: *CD14*, *TLR2*, *IFNAR*, *CXCL9*, *IL1RN* and *STAT1*, which were recently documented to be overexpressed particularly in M1 macrophages in both in vivo and in vitro models of macrophage specialization [14]. The transient silencing of catalase in non-stimulated cells resulted in the substantial increase in transcription of most considered genes (Figure 1E–G, Figure S1), whereas in combination with LPS only *IFNAR* responded with the additional enhancement of mRNA, suggesting the synergistic effect of the catalase silencing and M1 polarization. The lack of considerable LPS-induced augmentation of the expression of M1 markers, which are suppressed by catalase in non-specialized cells, may indicate that M1 polarization-associated drop in catalase abundance possibly allows or at least facilitates their increased transcription in response to LPS.

### 3.2. Repression of CAT Transcription in Response to TLR4 Activation Is Associated with the Formation of RNA Polymerase Pausing Complex

**Figure 2:**
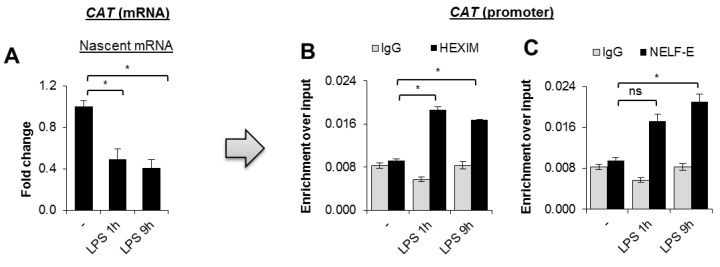
*CAT* repression is associated with the formation of POL2RA pausing complex in response to macrophage activation with LPS. (**A**) Newly minted RNA of *CAT* was captured by Click-iT chemistry after macrophage treatment with LPS for 1 and 9 h. (**B,C**) RNA polymerase II pausing in response to TLR4 stimulation was monitored by measuring HEXIM and NELF-E recruitment to the *CAT* promoter by ChIP-qPCR. Bars in the figures represent mean ± standard error of the mean (SEM). One-way analysis of variance (ANOVA) was carried out in GraphPad Prism 5 to compare means in several groups and the differences between each two groups were tested with the Tukey post hoc test (marked with * when *p* < 0.05 and with ns when *p* > 0.05).

Since the decline of the mRNA level may be caused by gene repression and degradation of the transcript (miR-30b was reported to inhibit the expression of endogenous catalase, at both the transcript and the protein levels in the human retinal pigment epithelial cell line exposed to a sublethal dose of hydrogen peroxide [15], we tested the rate of catalase mRNA de novo synthesis at the two time points—1 and 9 after induction of macrophage polarization with LPS. As shown in Figure 2A, activation of TRL4 caused an immediate response from the *CAT* regulatory element and a substantial reduction in the gene transcription, which was scarcely further inhibited at the later steps of macrophage polarization. This suggests that the observed decline in the mRNA level might be majorly regulated by the transcription yield.

Previous reports have described at least two regulatory elements that regulate *CAT* gene expression, in addition to proximal promoter [16]. To verify which of them is involved in the gene repression in pro-inflammatory macrophages, we first paid attention to the promoter adjacent to transcription start site, which is characterized by the presence of a CpG island (CGI). Knowing that *CAT* transcription is relatively quickly inhibited by LPS, and that RNA polymerase II (Pol II) elongation is terminated at the first few stable nucleosomes flanking the CGIs in response to intra- and extracellular signaling [17], we checked whether the TLR4 ligand induced formation of a Pol II pausing complex. As soon as 1 h after TLR4 stimulation, the two classic components of the pausing complex—HEXIM and NELF-E—were detected by ChIP real-time PCR at the *CAT* promoter (Figure 2B,C). No HEXIM and NELF-E enrichment was observed in the *CAT* exons 3 and 10 (Supplementary Figure S2; statistical analysis in Table S1). In the temporal aspect of gene repression, this agrees with the observed decrease in de novo mRNA synthesis and suggests that the proximal promoter might be involved in *CAT* repression in pro-inflammatory macrophages.

### 3.3. LSD1 Co-Operates with HDAC1 to Repress CAT in Macrophages Activated with LPS

**Figure 3:**
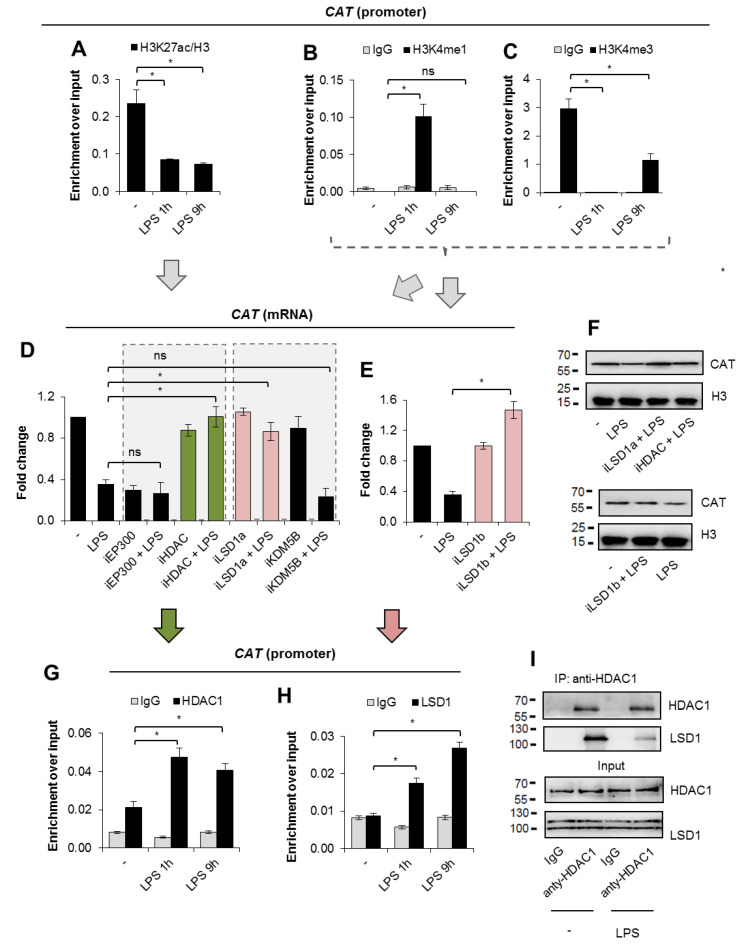
LSD1–HDAC1 complex suppresses *CAT* transcription during LPS-induced polarization of macrophages. ChIP-qPCR revealed the alteration in acetylation of H3K27 (**A**), monomethylation of H3K4 (**B**), and trimethylation of H3K4 (**C**) in the *CAT* promoter in response to macrophage stimulation with 10 ng/mL of LPS for 12 h. (**D**) The contribution of EP300, HDACs, LSD1, and KDM5B to LPS-induced *CAT* repression was studied by cell treatment with corresponding inhibitors (iEP300, 10 µM C646; iHDAC, 200 µM sodium butyrate; iLSD1a, 0.1 µM SP2509; iKDM5A, 5 µM PBIT) for 2 h prior to LPS. mRNA was collected after the next 24 h and *CAT* expression was quantified by real-time PCR. (**E**) The functional involvement of LSD1 activity in the loss of catalase was tested with one more inhibitor iLSD1b (0.1 µM GSK-LSD1). (**F**) iHDAC and iLSD1s protection from LPS-induced catalase protein decline was visualized by Western blot under the same experimental condition as in (**D**) and (**E**). The recruitment of HDAC1 (**G**) and LSD1 (**H**) to the *CAT* promoter was studied by ChIP-qPCR after macrophage treatment with LPS for 1 and 9 h. (**I**) The impact of LPS added to cells for 1 h on formation of LSD1–HDAC1 complexes was confirmed by immunoprecipitation of HDAC1, followed by detection of the interacting partners by Western blot. Western blots show representative images from three independent experiments. Bars in the figures represent mean ± standard error of the mean (SEM). One-way analysis of variance (ANOVA) was carried out in GraphPad Prism 5 to compare means in several groups and the differences between each two groups were tested with the Tukey post hoc test (marked with * when *p* < 0.05, with ns when *p* > 0.05).

Knowing that LPS induces alteration in the chromatin architecture at the CAT proximal promoter, we searched for the likely changes in the profile of histone post-translational modifications in the indicated region. We started analysis from the quantification of transcription-promoting histone marks, which were expected to be decreased once gene suppression was driven by the epigenetic mechanism. A substantial drop in histone acetylation was observed as soon as 1 h after macrophage stimulation with the TLR4 ligand, and the low level was maintained until the later time points (Figure 3A). Surprisingly, the removal of H3K4me3 and the emergence of H3K4me1 were transient (Figure 3B,C). This suggests that the decline in histone acetylation, which favors polymerase pausing, take place at the beginning of macrophage polarization to the pro-inflammatory phenotype [18]. However, paused Pol II maintains promoter regions in an accessible chromatin conformation due to the histone H3K4 methylation marks, which might be inserted, at least in part, by the SET complexes associated with the Ser-5 phosphorylated CTD of Pol II [19].

In the search for chromatin remodeling enzymes, which are possibly involved in *CAT* repression, we took advantage of the above-described observation on alterations in histone acetylation and H4K4 methylation. First, we took into consideration EP300, a histone acetyltransferase that activates transcription of a great majority of genes, and the HDAC family of histone deacetylases—enzymes that determine histone acetylation status. The inhibitor of EP300 (iEP300) alone declined *CAT* transcription comparably to LPS, and the synergistic effect of the two *CAT*-repressing agents was not observed, suggesting that EP300 acts as a *CAT* activator in M0 macrophages (Figure 3D). Pan-HDAC inhibitor (iHDAC) effectively restrained gene suppression, indicating that at least one member of the HDAC family contributed to LPS-induced limitation of transcription. The same approach was employed in testing the role of H3K4 demethylases in the *CAT* promoter’s response to TLR4 activation. Therefore, we pretreated macrophages with the inhibitor of lysine-specific histone demethylase 1 (iLSD1a, 0.1 µM SP2905), which demethylates mono- and dimethylated lysines 4 and 9, specifically at histone 3, and with the inhibitor of lysine demethylase 5B (iKDM5B), which demethylates tri-, di-, and monomethylated lysine 4 of histone H3. iLSD1a—SP2905 attenuates the binding of LSD1 to corepressor protein CoREST that is necessary for LSD1 to demethylate nucleosomes [20]. Since hydroxyphenyl hydrazone moiety is well known to feature lots of non-specific effects [21], which arise also by off-target, we employed another, irreversible, mechanism-based inhibitor of LSD1—GSK-LSD1 (iLSD1b) that is >1000-fold selective over the closely related FAD-utilizing enzymes LSD2, MAO-A, and MAO-B [22]. As shown in Figure 3E, iLSD1b acted equally to iLSD1a and prevented *CAT* transcription from LPS-induced repression. The protective activity of pan-HDAC and LSD1 inhibitors was also confirmed by Western blot, because all three inhibitors comparably maintained high levels of the enzyme in the pro-inflammatory macrophages (Figure 3F, Figure S1). Although KDM5B is, similarly to LSD1, involved in the removal of methyl groups from histone H3, we did not observe any considerable effect of KDM5B inhibition on the catalase transcription. This suggests that KDM5B might not be responsible for the LPS-induced decrease in catalase expression.

The analysis of *CAT* promoter by ChIP real-time PCR supported the conclusions arising from the previous inhibitor-based approach and disclosed the recruitment of LSD1 (Figure 3G) and HDAC1 (Figure 3H) as soon as 1 h after macrophage stimulation with LPS. This observation agrees with previous reports, which described co-operativity between LSD1 and HDAC1 within the CoREST complexes [23]. Bearing in mind that HDAC1 and LSD1 occur at the promoter of the studied gene, we asked if such protein heterodimers are formed in response to TLR4 activation. Immunoprecipitation of HDAC1 revealed a physical interaction with demethylase in unstimulated cells (Figure 3I, Figure S1). The demethylase was visibly reduced in HDAC1 pull-downs from LPS-stimulated cells, thereby suggesting that (**a**) pre-existing HDAC1–LSD1 complexes disintegrate 24 h after macrophage stimulation with LPS, or (**b**) LSD1 is selectively degraded in the above-mentioned complexes, because the total intracellular level of the enzyme (Figure 3I—input) remains the same in LPS-treated and -untreated cells.

### 3.4. HDAC Alone Maintains CAT Repression in M1-Polarized Macrophages

**Figure 4:**
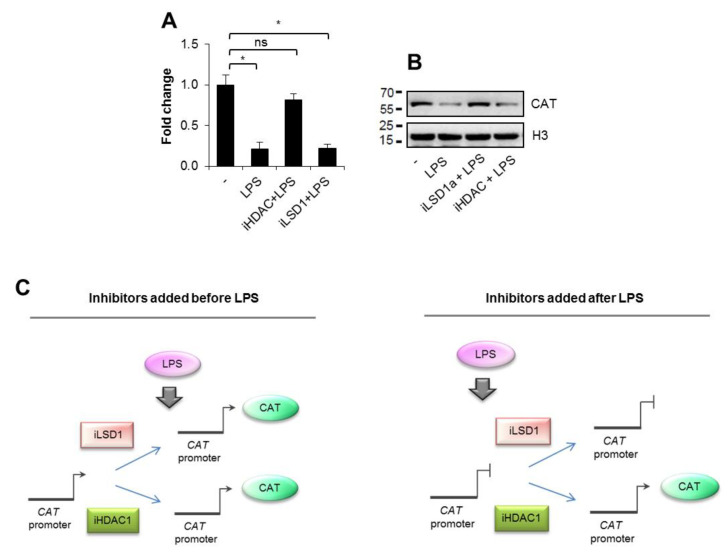
The inhibition of HDACs, but not LSD1, reverses *CAT* suppression in pro-inflammatory macrophages. iHDAC (200 µM sodium butyrate) and iLSD1a (0.1 µM SP2509) were used to restore transcription of *CAT* in pro-inflammatory macrophages. The two inhibitors were added 24 h after cell stimulation with LPS (10 ng/mL) for another 24 h. The mRNA level (**A**) was quantified by real-time PCR, whereas protein level (**B**) by Western blot. (**C**) The scheme shows the difference in the iLSD1 effect on the *CAT* expression depending on the macrophage phenotype. The treatment of non-polarized phagocytes with both inhibitors prevents *CAT* repression induced by LPS (left panel), but only iHDAC1 restores *CAT* transcription in M1 macrophages (right panel). Western blots show representative images from three independent experiments. Bars in the figures represent mean ± standard error of the mean (SEM). One-way analysis of variance (ANOVA) was carried out in GraphPad Prism 5 to compare means in several groups and the differences between each two groups were tested with the Tukey post hoc test (marked with * when *p* < 0.05, with ns when *p* > 0.05).

Knowing the profile of histone remodeling enzymes that bring about catalase deficiency in LPS-stimulated macrophages, we tested whether their inhibitors can be used to restore catalase expression after suppression. Therefore, we first allowed M0 macrophages to polarize by incubating them with LPS for 24 h, and then treated them with the inhibitors for another 24 h. iHDAC, but not iLSD1a, reverted gene repression in pro-inflammatory macrophages developed after cell activation with the TLR4 ligand, as well as increased mRNA and the enzyme protein to a level comparable to M0 macrophages (Figure 4A,B, Figure S1). It suggests that in LPS-induced pro-inflammatory macrophages, a decline in promoter acetylation plays a superior role in the reduction in *CAT* transcription and that LSD1 activity promotes the gene repression only at the initial step. This also corresponds with the results shown in Figure 3B,C, where the initial loss of transcription-promoting trimethylation of H3K4 was reconstituted at a later step of macrophage polarization. Although LSD1 remained associated with the gene promoter in pro-inflammatory macrophages, its role in gene suppression was taken over by HDAC1, which maintained deacetylated nucleosomes in the *CAT* promoter (Figure 3A).

### 3.5. LSD1 Inhibition Interferes with the HDAC1 Recruitment to the CAT Promoter and Protects the Gene from LPS-Triggered Repression

**Figure 5:**
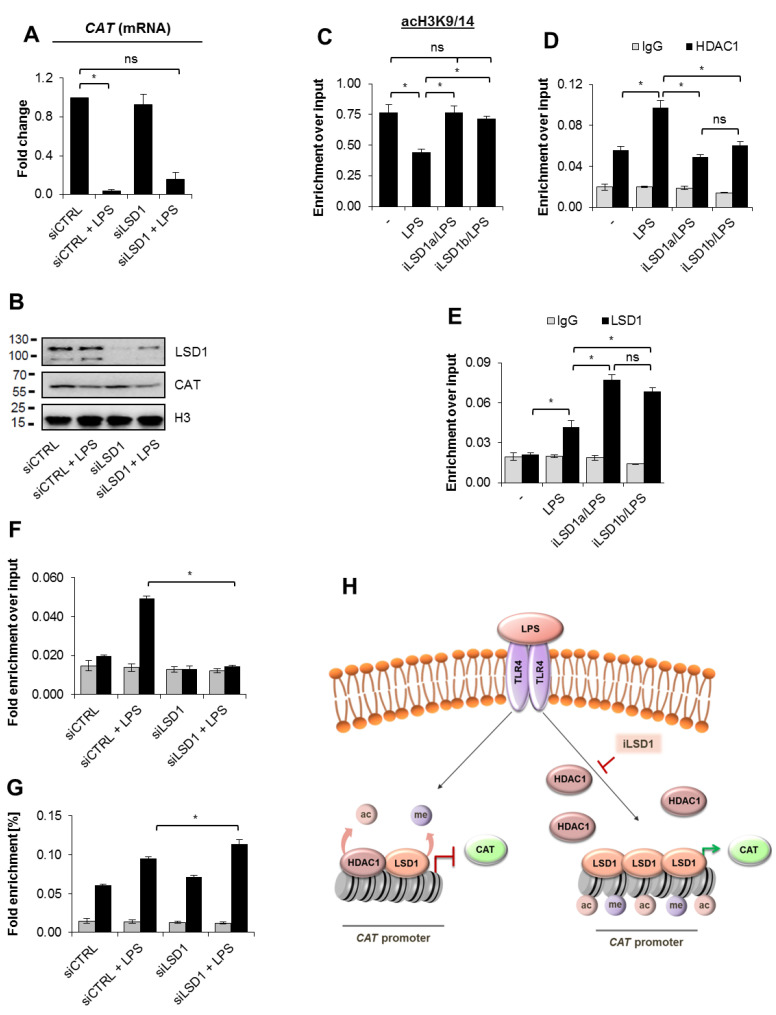
LSD1 activity allows for HDAC1 recruitment to the *CAT* promoter and the gene repression in cells stimulated with LPS. The impact of LSD1 deficiency on the *CAT* transcription was studied in LSD1 transient knock downs (siLSD1) and corresponding control cells (siCTRL) by real-time PCR (**A**) and Western blot (**B**) 48 h after cell transfection. The effect of LSD1 inhibition on chromatin composition (**C**,**D**) and histone acetylation (**E**) in cells activated with LPS for 12 h was studied by ChIP-qPCR. iLSD1a (0.1 µM SP2509) and iLSD1b (0.1 µM GSK-LSD1) were added for 2 h prior to cell treatment with 10 ng/mL LPS. The recruitment of LSD1 and HDAC1to the *CAT* promoter in normal and LSD1-deficient macrophages stimulated with LPS was monitored by ChIP-qPCR (**F**,**G**). (**H**) Graphical presentation of the possible cross-talk between LSD1 activity, HDAC1 recruitment to the *CAT* promoter, chromatin structure (histone acetylation) and the *CAT* transcription. Inhibition of LSD1 activity enhances the binding of demethylase but interferes with the recruitment of HDAC1 upon macrophage stimulation with LPS. Western blots show representative images from three independent experiments. Bars in the figures represent mean ± standard error of the mean (SEM). One-way analysis of variance (ANOVA) was carried out in GraphPad Prism 5 to compare means in several groups and the differences between each of the two groups were tested with the Tukey post hoc test (marked with * when *p* > 0.05, with ns when *p* > 0.05).

To further study the role of LSD1 in the modification of the chromatin structure at the catalase promoter we tested the impact of LSD1 protein deficiency at the mRNA level of hydrogen peroxide decomposing enzyme. We transiently silenced LSD1 with siRNA and stimulated cells with bacterial endotoxin after 48 h. Surprisingly, the lack of LSD1 protein did not rescue suppression of catalase (Figure 5A,B, Figure S1). It suggests that upon inhibition of LSD1 activity the enzyme protein prevents *CAT* repression in an unknown fashion, whereas the simultaneous deficiency of LSD1 activity and protein allows for the proper signal transmission from TLR4 to the gene promoter. In search for the possible contribution of LSD1 protein in the absence of its activity to the maintenance of *CAT* transcription we first checked if LSD1 inhibition affects histone acetylation status since this modification emerged responsible for the *CAT* repression in the polarized macrophages (Figure 4A,B). Interestingly, both LSD1 inhibitors protected the gene promoter from acetylation decline in LPS-treated cells (Figure 5C). This can be possibly explained by the reduced HDAC1 recruitment to chromatin in the presence of LSD1 inhibitors (Figure 5D). The iLSD1-induced chromatin alteration in LPS-treated cells were followed by the enhanced LSD1 occurrence at the gene promoter (Figure 5E). Moreover, LSD1 silencing slightly enhanced HDAC1 recruitment to the *CAT* promoter (Figure 5F,G), thereby confirming the role of LSD1 in HDAC1 interaction with the studied region of chromatin. The subtle increase in HDAC1 occupancy at the *CAT* promoter upon LSD1 protein deficiency in macrophages stimulated with LPS may indicate near chromatin dissipation with histone deacetylase in cells with physiological level of LSD1. The innate LSD1 recruitment to chromatin merely interferes with HDAC1 occurrence and nucleosome deacetylation, but the iLSD1-induced increase in chromatin occupancy by LSD1 inhibits HDAC1 interaction with the *CAT* promoter. The possible functional link between LSD1 activity, its interaction with the *CAT* promoter, the binding of HDAC1 and covalent modification of transcription-regulating histones are presented in Figure 5H.

### 3.6. The Maintenance of Catalase Level in Polarizing Macrophages Lessens Expression of Some Pro-Inflammatory Markers

**Figure 6:**
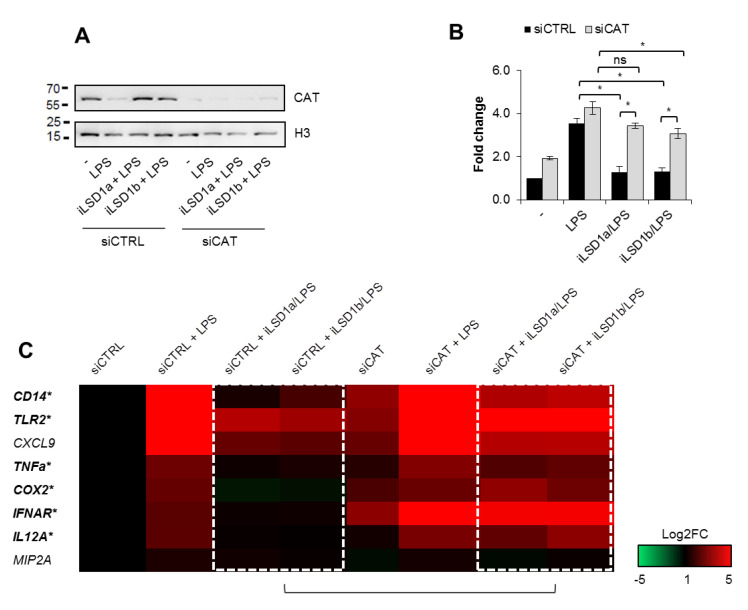
LSD1-induced suppression of catalase facilitates the obtention of some pro-inflammatory markers in M1-polarizing cells. To test the possible role of catalase in M1 polarization the LSD1-induced *CAT* repression was prevented with iLSD1a and iLSD1b (0.1 µM SP2509, 0.1 µM GSK-LSD1, respectively; added for 2 h prior to LPS), and the mRNA of some M1 markers was quantified (real-time PCR) and compared between cells with the maintained and decreased level of catalase (siCTRL vs. siCAT). (**A**) The efficiency of catalase silencing by siCAT in the presence and absence of iLSD1a/b was tested by Western blot 48 h after cell transfection with siRNA. Inhibitors were added 46 h after cell transfection and for 2 h prior to LPS (10 ng/mL). Cell lysates were collected 24 h after cell stimulation with LPS. (**B,C**) The same experimental setting were applied to testing some cytokines and other M1 markers. (**B**) IL1β is shown as an example. (**C**) Bolded gene names marked with stars indicate genes repressed by catalase during M1 polarization (significant difference in the mean when *p* < 0.05 between siCTRL + iLSD1a/LPS and siCAT + iLSD1a/LPS, as well as between siCTRL + iLSD1b/LPS and siCAT + iLSD1b/LPS). Western blots show representative images from three independent experiments. Bars in the figures represent mean ± standard error of the mean (SEM). One-way analysis of variance (ANOVA) was carried out in GraphPad Prism 5 to compare means in several groups and the differences between each two groups were tested with the Tukey post hoc test (marked with * when *p* > 0.05, with ns when *p* > 0.05).

To verify the hypothesis on the possible suppressive role of catalase in the transcription control of some LPS-induced pro-inflammatory cytokines and M1 surface markers in polarizing cells, we made use of LSD1 inhibitors to prevent catalase decline (Figure 6A, Figure S1). Since LSD1 directly affects transcription of NF-κB-dependent genes by demethylating and, thus, enhancing p65 protein stability [24], we compared the effect of LSD1 inhibitors on the M1 marker transcription in the siCTRL and siCAT background. Western blot in Figure 6A confirmed that the transient catalase silencing preserved catalase increase in iLSD1-treated cells. Cells activated by LPS in the presence of iLSD1a and iLSD1b were characterized by the reduced mRNA level of genes negatively controlled by catalase in LPS non-stimulated cells (Figure 1F,G). Transcription of *IL1β*, *TNFα*, *COX2*, *CD14* and *TLR2* lost sensitivity to LSD1 inhibition in catalase deficient cells (Figure 6B,C). The silencing of catalase prevented the decline in the LPS-induced gene transcription caused by iLSD1a/b. These results indicate that LSD1 activation after TLR4 interaction with bacterial ligand facilitates the obtention of pro-inflammatory phenotype by suppressing catalase transcription.

## 4. Discussion

Lysine-specific histone demethylase 1A (LSD1) demethylates both “Lys-4” (H3K4me) and “Lys-9” (H3K9me) of histone H3, thereby acting as a transcriptional corepressor or coactivator, respectively [25]. The nature of the catalytic activity of LSD1 makes this enzyme directly involved in the regulation of intracellular redox processes since flavin adenine dinucleotide (FAD)-dependent amine oxidation mediated by this enzyme generates detectable ROS as a byproduct of its chromatin remodeling activity during, for example, the initial DNA damage response [26]. Our current and previous papers describe the contribution of the demethylase to the adjustment of the enzymatic redox response during the polarization of human macrophages. Upon their stimulation with TLR4 ligand, LSD1 represses both *SOD2* and *CAT*, which decompose the mitochondrial superoxide anion and hydrogen peroxide, respectively [7]. Despite their repression, pro-inflammatory macrophages reveal relatively strong resistance to oxidative stress, and LSD1 inhibition merely increases their viability [7]. Although the effect of enzyme inhibition on macrophages’ response to toxic doses of exogenous hydrogen peroxide is still considerable, the major role of shifting redox balance to a pro-oxidative condition rather renders M1 macrophages to pathogen killing and regulates inflammatory responses. The superoxide anion, together with the iNOS-derived NO, serves as a substrate for the synthesis of the powerful oxidant peroxynitrite, whereas hydrogen peroxide is utilized by myeloperoxidase to generate hypochlorous acid. Pro-inflammatory cytokines and ROS stimulate the production of each other [27]; therefore, by targeting *SOD2* and *CAT* transcription, the LSD1 inhibitors may affect various intracellular processes and may unveil immunomodulatory effects in polarized macrophages. Additionally, by directly modulating the transcription of *IL1β*, *TNFa*, and *IL6*, LSD1 acts as a critical regulator of mammalian hematopoiesis and as a negative regulator of the response to inflammation in hematopoietic stem cells during endotoxic shock [28,29]. The demethylation of p65 by LSD1 was shown to be critical for the inflammatory response in vivo since it enhances p65 protein stability [24]. In line with above-described findings, our current paper describes another aspect regarding LSD1 role in pro-inflammatory response, namely repression of catalase that serves as a negative regulator of some M1-specific genes, including pro-inflammatory cytokines. Thus, LSD1 can possibly promote dually the M1 macrophage specialization by directly modifying p65 and by reducing catalase abundance.

The co-occurrence of LSD1 and HDAC1 at the gene promoters and the following gene suppression is assigned in the literature to the formation of CoREST-based multi-subunit co-repressive complexes, where RE1 silencing transcription factor (REST) cooperates with the corepressor of REST (CoREST), LSD1, and HDAC1/2. The precise composition of these complexes differs depending on cell type, but other core subunits, such as CtBP1, ZNF217, BHC80, and BRAF35, were identified [30]. As long as HDAC1 can be recruited to chromatin with other proteins to remove acetyl groups from chromatin-bound factors and nucleosomes, the interaction with CoREST was deemed necessary for LSD1 to demethylate H3K4. Indeed, TRL4 stimulation with LPS led to co-recruitment of LSD1 and HDAC1 to the *CAT* promoter, and simultaneous histone demethylation and deacetylation. However, the inhibition of LSD1 activity led to substantial enrichment of the enzyme at the gene promoter and prevented HDAC1 binding, thereby rescuing nucleosome acetylation and catalase transcription in cells treated with LPS. Activity of LSD1 turns out as a factor limiting the physical interaction of the enzyme with chromatin. Even more surprising was the LSD1 enrichment at the CAT promoter after attenuation of LSD1 binding to CoREST with SP2509, particularly when the CoREST SANT2 domain was proposed to function as a molecular bridge that connects LSD1 to its nucleosomal substrates [31]. Very recent findings paid attention to nonenzymatic functions of LSD1 that emerged critical for its activities [32]. In the referred model of retinoic acid-induced differentiation of acute myeloid leukemia cells LSD1 played a scaffolding role, and LSD1 inhibition impeded the interaction between LSD1 and GFI1, but not with HDAC1/2 and RCOR1. Moreover, LSD1 inhibitors were shown to target catalytic and noncatalytic functions of LSD1. The question on the mechanism that drives HDAC1 recruitment to the *CAT* promoter during pro-inflammatory macrophage polarization remains open. In LSD1 deficient cells HDAC1 binds and represses *CAT* promoter, but it equally occurs in LSD1 proficient cells as long as the enzyme is active and removes the methyl groups from histone tails. Therefore, the status of histone methylation does not seem to be crucial for HDAC1-mediated gene inactivation. The considerable increase in LSD1 occurrence on the chromatin upon the enzyme inhibition may suggest the possible interference with the HDAC1 binding, but further experiments are needed to conclude on the mutual interdependence between LSD1 and HDAC1 activity and recruitment to the chromatin.

## 5. Conclusions

In conclusion, LSD1 promotes the macrophage pro-inflammatory specialization by repressing catalase in response to activation of TLR4 receptors with LPS. Hydrogen peroxide-decomposing enzyme impedes transcription of the subset of pro-inflammatory cytokines and M1-specific surface markers; therefore, targeting of LSD1 activity may be considered as the immunosuppressive treatment for some pathological conditions associated with hyperactivation of pro-inflammatory macrophages.

## Figures and Tables

**Table 1:** Primers used in real-time PCR reaction.

Genes	Forward (5′–3′)	Reverse (5′–3′)
* CAT *	ACTTTGAGGTCACACATGACATT	CTGAACCCGATTCTCCAGCA
* ACTB *	TGGCACCCAGCACAATGAA	CTAAGTCATAGTCCGCCTAGAAGCA
* GAPDH *	GGAGTCAACGGATTTGGTCGTA	GGCAACAATATCCACTTTACCA
* B2M *	GACTTGTCTTTCAGCAAGGA	ACAAAGTCACATGGTTCACA
* TNFα *	GGAGAAGGGTGACCGACTCA	GAAACGGCTCAGACCCGT
* COX2 *	GAATCATTCACCAGGCAAATTG	TCATGTCTTTCATAGTGTCCGAAGGT
* IL12A *	CTCCTGGACCACCTCAGTTTG	TTACAAGGGTACGGAAGTGG
* MIP2A *	CGCCCAAACCGAAGTCAT	TTTCTACGACTTTTTACCGTTTAG
* IL1β *	ACGAATCTCCGACCACCACT	CAGTCAACAACACCGGTACC

## Data Availability

Raw data are included in Table S1.

## Supplementary Materials

The following are available online at https://www.mdpi.com/article/10.3390/cells10092465/s1, Figure S1: Full Western blot images, Figure S2: The occurrence of POLR2 pausing complex in the *CAT* exons, Table S1: Raw data and statistical analysis.
