# Supplementary material for: LSD1 Facilitates Pro-Inflammatory Polarization of Macrophages by Repressing Catalase
Source: Cells. 2021 Sep 18;10(9):2465. doi: 10.3390/cells10092465 (PMC8469135; doi:10.3390/cells10092465)
Supplement: Supplementary file 1 [file cells-10-02465-s001.zip › Figure S1.pdf]

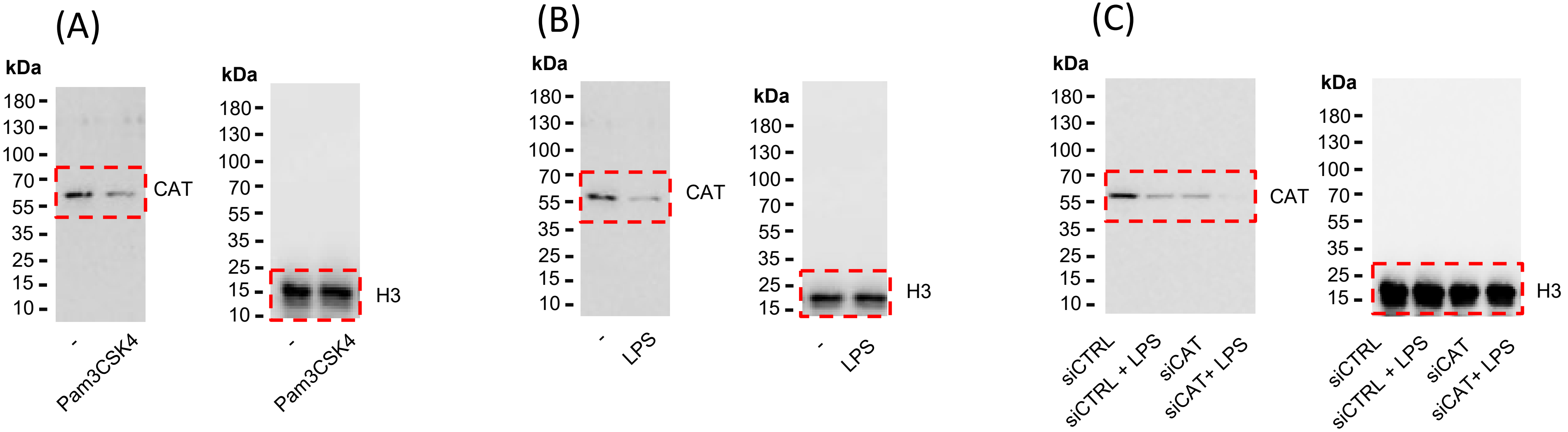

(A)
kDa
180
130
100
70
55
35
25
15
10
CAT
H3
-
Pam3CSK4
(B)
LPS
(C)
siCTRL
siCTRL + LPS
siCAT
siCAT+ LPS

(D)

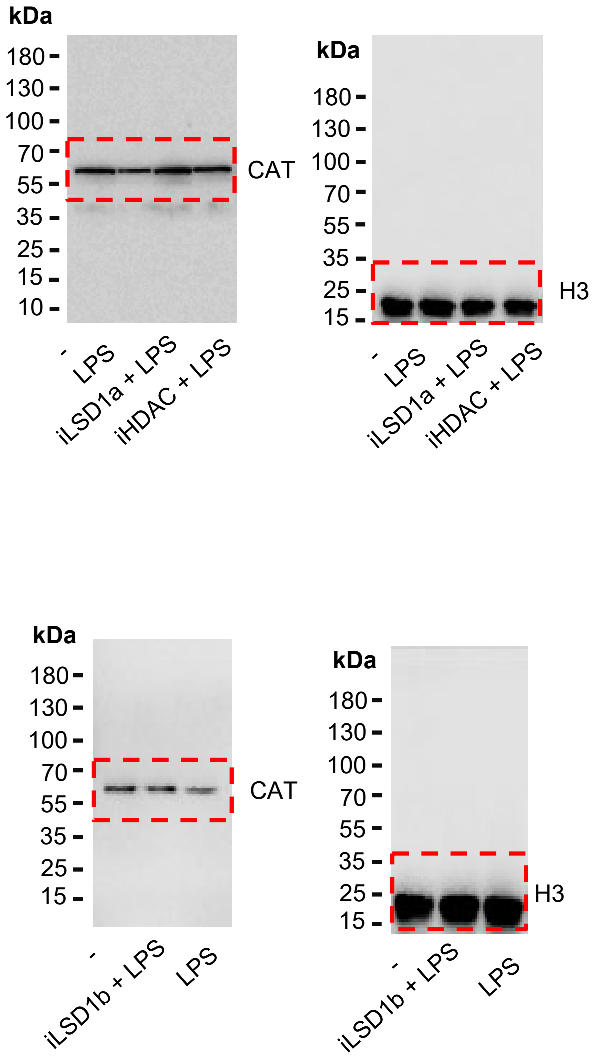
kDa
180
130
100
70
55
35
25
15
10
CAT
H3
-
LPS
iLSD1a + LPS
iHDAC + LPS
iLSD1b + LPS

(E)

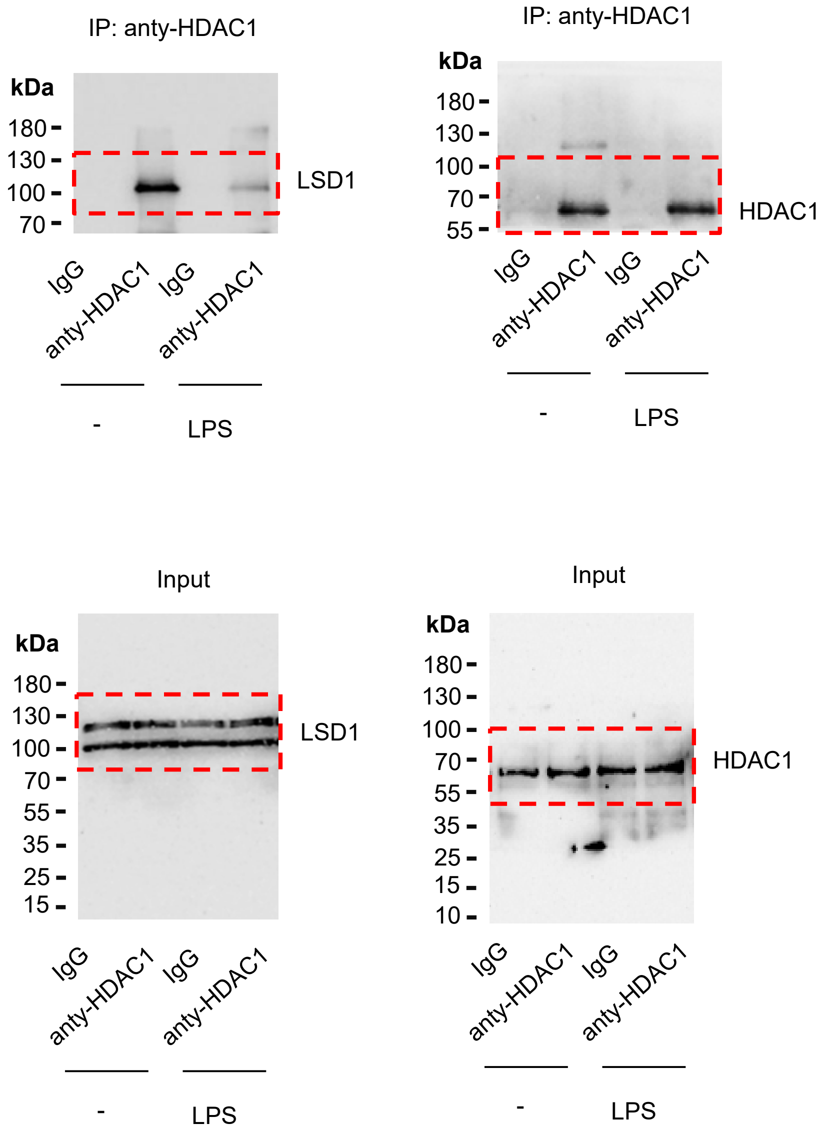
IP: anty-HDAC1
Input
kDa
180
130
100
70
55
35
25
15
10
LSD1
HDAC1
IgG
anty-HDAC1
-
LPS

(F)

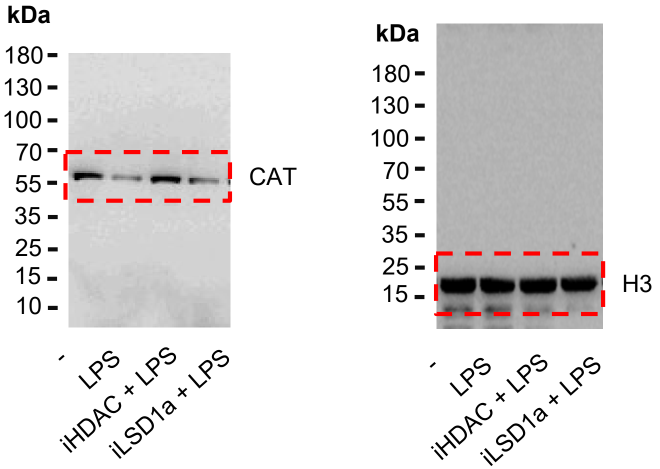
kDa
180
130
100
70
55
35
25
15
10
CAT
-
LPS
iHDAC + LPS
iLSD1a + LPS
kDa
180
130
100
70
55
35
25
15
H3
-
LPS
iHDAC + LPS
iLSD1a + LPS

(G)

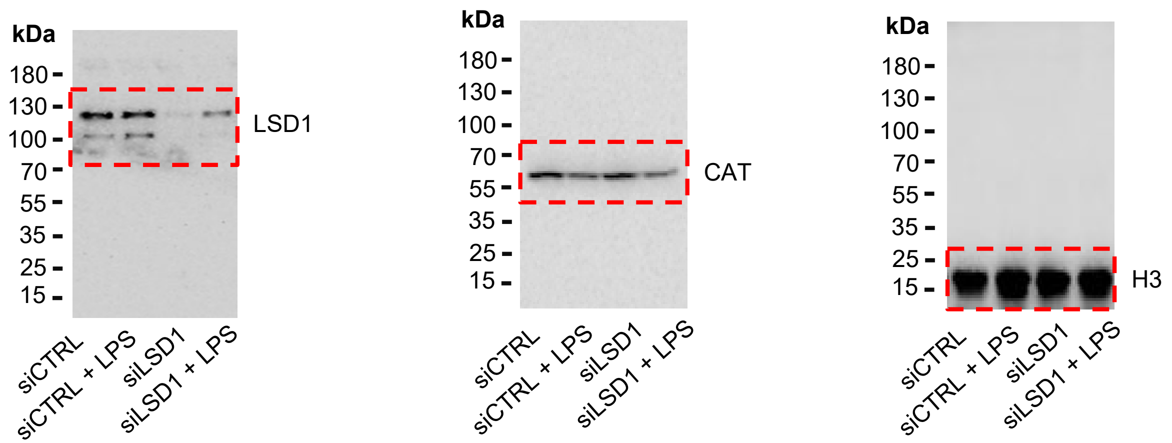
kDa
180
130
100
70
55
35
25
15
LSD1
siCTRL
siCTRL + LPS
siLSD1
siLSD1 + LPS
kDa
180
130
100
70
55
35
25
15
CAT
siCTRL
siCTRL + LPS
siLSD1
siLSD1 + LPS
kDa
180
130
100
70
55
35
25
15
H3
siCTRL
siCTRL + LPS
siLSD1
siLSD1 + LPS

(H)

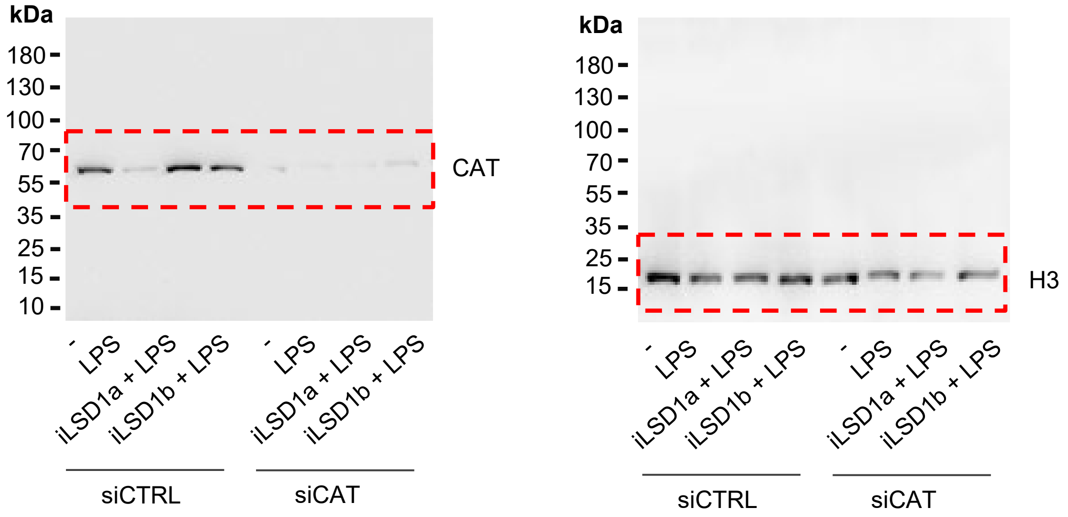

Figure S1. (**A**). Supplementary figure for Figure 1A. (**B**). Supplementary figure for Figure 1C. (**C**). Supplementary figure for Figure 1E. (**D**). Supplementary figure for Figure 3F. (**E**). Supplementary figure for Figure 3I. (**F**). Supplementary figure for Figure 4B. (**G**). Supplementary figure for Figure 5B. (**H**). Supplementary figure for Figure 5A.
