# Supplementary figures and images for: LSD1 Facilitates Pro-Inflammatory Polarization of Macrophages by Repressing Catalase
Source: Cells. 2021 Sep 18;10(9):2465. doi: 10.3390/cells10092465 (PMC8469135; doi:10.3390/cells10092465)

Exon 3

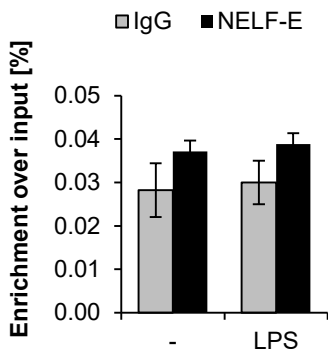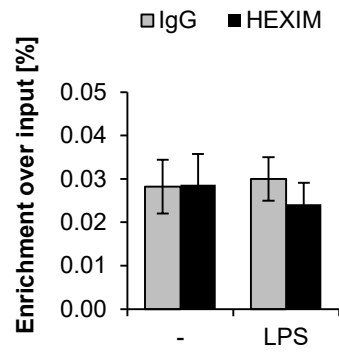

Exon 10

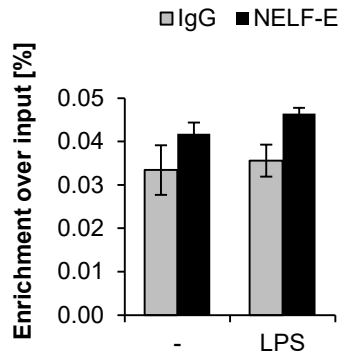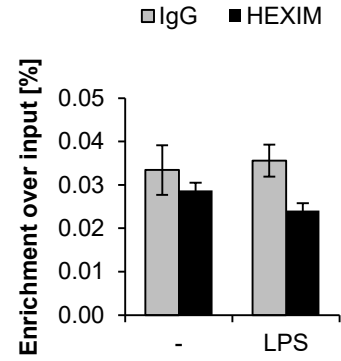

Supplement: Supplementary file 1 [file cells-10-02465-s001.zip › Figure S2.pdf]
